# Definitions of bronchopulmonary dysplasia and long-term outcomes of extremely preterm infants in Korean Neonatal Network

**DOI:** 10.1038/s41598-021-03644-7

**Published:** 2021-12-21

**Authors:** Ga Won Jeon, Minkyung Oh, Yun Sil Chang

**Affiliations:** 1 Department of Pediatrics, Inje University Busan Paik Hospital, Inje University College of Medicine, Busan, South Korea; 2 Department of Pharmacology, Inje University College of Medicine, Busan, South Korea; 3 Department of Pediatrics, Samsung Medical Center, Sungkyunkwan University School of Medicine, 81 Irwon-Ro, Gangnam-Ku, Seoul, 06351 South Korea

**Keywords:** Neonatology, Paediatric research, Preterm birth

## Abstract

New definitions for bronchopulmonary dysplasia (BPD) have recently been suggested, and an accurate diagnosis, including severity classification with proper definition, is crucial to identify high-risk infants for appropriate interventions. To determine whether recently suggested BPD definitions can better predict long-term outcomes of BPD in extremely preterm infants (EPIs) than the original BPD definition, BPD was classified with severity 1, 2, and 3 using three different definitions: definition A (original), National Institute of Child Health and Human Development (NICHD) definition in 2001; definition B, the modified NICHD 2016 definition (graded by the oxygen concentration and the respiratory support at 36 weeks’ postmenstrual age [PMA]); and definition C, the modified Jensen 2019 definition (graded by the respiratory support at 36 weeks’ PMA). We evaluated 1050 EPIs using a national cohort. Whereas EPIs with grade 2 or 3 BPD as per definition A did not show any increase in the risk, EPIs with BPD diagnosed by definition B and C showed significantly increased risk for poor outcomes, such as respiratory mortality and morbidities, neurodevelopmental delay, and growth restriction at 18–24 months of corrected age. The recently suggested definition and severity grading better reflects long-term childhood morbidities than the original definition in EPIs.

## Introduction

Bronchopulmonary dysplasia (BPD) is one of the most serious complications in preterm infants. The incidence of BPD has not decreased, having even increased, with the increased survival of extremely preterm infants (EPIs) with gestational age (GA) of less than 28 weeks who are at great risk for the development of BPD^1,2^. BPD has long-term adverse effects on respiratory, neurodevelopmental, and growth outcomes, and contributes to early mortality as well^3,4^. To improve long-term outcomes of BPD, early exact diagnosis and severity grading of BPD are crucial to better predict long-term outcomes and identify high-risk infants requiring early intervention to decrease the chronic comorbidities of BPD. The definition of BPD has been revised several times over the past 50 years since its first description^5–8^. The task of defining BPD has become more challenging as more EPIs survive, and new respiratory support options are developed. The most widely used definition was the one described in the National Institute of Child Health and Human Development (NICHD) workshop in 2001^9^. However, preterm infants with mild BPD, as per this definition, were not at increased risk of long-term morbidities^10,11^, suggesting that it is inappropriate to trap mild BPD as “BPD.” In addition, some infants with severe BPD require respiratory support for months or even years^12^, suggesting the need to stratify severe BPD into subgroups. Moreover, newly developed respiratory support is not classified by this definition, and preterm infants who die of respiratory failure before 36 weeks’ postmenstrual age (PMA), the most severe form of BPD, are not diagnosed with BPD according to this definition.

In order to address these problems and to better predict long-term outcomes, the NICHD workshop in 2016 suggested a new definition of BPD^13^ without precondition of oxygen or respiratory support for the first 28 days of life. BPD was graded according to oxygen concentration and respiratory support assessed only at 36 weeks’ PMA by this definition. Another definition was suggested by Jensen et al. in 2019^14^. This definition also does not require a precondition of oxygen or respiratory support in the first 28 days of life. BPD was graded according to the respiratory support assessed at 36 weeks’ PMA regardless of oxygen concentration (grade 1: nasal cannula ≤ 2 L/min; grade 2, non-invasive ventilator [NIV] (including nasal cannula > 2 L/min); and grade 3, invasive ventilator [IV]). This definition could better predict long-term outcomes and identify infants at the greatest risk for poor outcomes^15,16^. However, a number of BPDs are still defined by the NICHD 2001 definition, so it is necessary to verify the validity of recently suggested definitions in a large cohort of EPI with the highest risk for the development of BPD. To better predict long-term outcomes, we compared long-term morbidities of preterm infants with BPD defined by recently suggested definitions with those of the original NICHD 2001 definition.

In this study, we redefined the cohort of EPIs who survived 36 weeks’ PMA by three definitions of BPD: original NICHD 2001, modified NICHD 2016, and modified Jensen 2019. We then analyzed the risks for poor respiratory, neurodevelopmental, and growth outcomes at corrected age (CA) 18–24 months according to the severity of BPD according to each definition.

## Results

### Characteristics of patients (Table 1)

A total of 1707 very low birth weight infants (VLBWIs) who were registered in the Korean Neonatal Network (KNN), were identified initially. Mean GA was 25.7 ± 1.2 weeks, and mean birth weight (BWt) was 875.5 ± 193.7 g. Their characteristics are shown in Supplementary Table 1.

The rates of follow-up at 18–24 months of CA were 1050/1481 (70.9%) who were finally enrolled in the study. Mean GA was 25.7 ± 1.2 weeks, and mean BWt was 868.4 ± 195.4 g. Infants with GA of 23, 24, 25, 26, and 27 weeks were 65/1050 (6.2%); 119/1050 (11.3%); 254/1050 (24.2%); 267/1050 (25.4%); and 345/1050 (32.9%), respectively. Infants with BWt of < 500 g, 500 ~  < 750 g, 750 ~  < 1000 g, and 1000 ~  < 1500 g were 30/1050 (2.9%); 264/1050 (25.1%); 478/1050 (45.5%); and 278/1050 (26.5%), respectively. Small for gestational age (SGA) was 74/1050 (7.0%), and Apgar scores at 1 min and 5 min were 3.8 ± 1.8, and 6.1 ± 1.8, respectively. The rates of maternal antenatal steroid therapy and chorioamnionitis were 867/1032 (84.0%) and 441/924 (47.7%), respectively. The rates of maternal gestational diabetes mellitus and pregnancy-induced hypertension were 67/1042 (6.4%) and 93/1034 (9.0%), respectively (Table 1).

**Table 1:** Characteristics of patients followed up at 18–24 months of corrected age.

	Total N = 1050
Gestational age, weeks	25.7 ± 1.2
23, n (%)	65/1050 (6.2%)
24, n (%)	119/1050 (11.3%)
25, n (%)	254/1050 (24.2%)
26, n (%)	267/1050 (25.4%)
27, n (%)	345/1050 (32.9%)
Birth weight, g	868.4 ± 195.4
< 500 g, n (%)	30/1050 (2.9%)
500 ~ < 750 g, n (%)	264/1050 (25.1%)
750 ~ < 1000 g, n (%)	478/1050 (45.5%)
1000 ~ < 1500 g, n (%)	278/1050 (26.5%)
Male, n (%) (sex)	548/1050 (52.2%)
Small for gestational age, n (%)	74/1050 (7.0%)
Apgar score at 1 min	3.8 ± 1.8
Apgar score at 5 min	6. 1 ± 1.8
Antenatal steroid therapy, n (%)	867/1032 (84.0%)
Maternal chorioamnionitis	441/924 (47.7%)
Maternal GDM	67/1042 (6.4%)
Maternal PIH	93/1034 (9.0%)

Values are expressed as mean ± standard deviation or number (%). GDM, gestational diabetes mellitus; PIH, pregnancy induced hypertension.

### Definitions for BPD assessed at 36 weeks’ PMA (Table 2)

BPD by definition B (modified NICHD 2016 definition) is graded according to oxygen concentration and respiratory support. BPD by definition C (modified Jensen 2019 definition) is graded according to respiratory support, regardless of oxygen concentration. Definitions B and C do not require the precondition of oxygen or respiratory support during the first 28 days of life.

**Table 2:** Definitions for bronchopulmonary dysplasia assessed at 36 weeks’ postmenstrual age.

	Definition A	Definition B	Definition C
Grade 1	(Treatment with O_2_, FiO_2_ > 21% for at least 28 days plus); breathing room air	O_2_ < 2L/min, FiO_2_ > 0.21 O_2_ ≥ 2L/min, FiO_2_ 0.21 NIV, FiO_2_ 0.21	O_2_ < 2L/min
Grade 2	O_2_, FiO_2_ 0.22–0.29	O_2_ ≥ 2L/min, FiO_2_ 0.22–0.29 NIV, FiO_2_ 0.22–0.29 IV, FiO_2_ 0.21	O_2_ ≥ 2L/min NIV
Grade 3	O_2_, FiO_2_ ≥ 0.3 Positive pressure ventilator	O_2_ ≥ 2L/min, FiO_2_ ≥ 0.3 NIV, FiO_2_ ≥ 0.3 IV, FiO_2_ > 0.21	IV

NIV, non-invasive ventilator; IV, invasive ventilator.

BPD by definition B (modified NICHD 2016 definition) is graded according to oxygen concentration and respiratory support. BPD by definition C (modified Jensen 2019 definition) is graded according to respiratory support, regardless of oxygen concentration. Definitions B and C do not require oxygen or respiratory support for the first 28 days of life.

### Agreement between three different definitions of BPD (Supplementary Table 2)

The frequencies of BPD were 1014 (96.8%) by definition A and 527 (50.3%) by definitions B and C. Almost all grade 1 BPDs by definition A (461) was allocated to no BPD according to definitions B and C (455). Most grade 2 BPDs by definition A (200) was allocated to grade 1 BPD according to definitions B (160) and C (158). A total number of 353 grade 3 BPDs by definition A was mainly distributed to grade 1, 73; grade 2, 121; and grade 3, 148 by definition B. Most of the grade 3 BPDs by definition A (353) was allocated to grades 2 (255) and 3 (82) by definition C.

### Mortality or morbidities due to respiratory causes (Table 3)

A total of 115 infants (11.0%, 115/1050) had mortality or morbidities due to respiratory causes at follow-up between 18 and 24 months of CA. Eight infants died due to respiratory causes between 36 weeks’ PMA and 18–24 months of CA. One hundred and five infants had at least three readmissions due to respiratory causes after discharge from the neonatal intensive care unit (NICU) prior to follow-up.

**Table 3:** Adjusted odds ratios using multivariable logistic regression to evaluate mortality or morbidities due to respiratory causes at 18–24 months of corrected age according to the severity of bronchopulmonary dysplasia (BPD) compared to no BPD.

	Definition A	Definition B	Definition C
Grade 1	0.65 (0.20, 2.11)	1.54 (0.91, 2.62)	1.53 (0.84, 2.78)
Grade 2	1.10 (0.33, 3.70)	2.28 (1.25, 4.15)	2.10 (1.27, 3.48)
Grade 3	1.83 (0.56, 5.98)	3.40 (1.98, 5.83)	4.41 (2.37, 8.23)

Values are expressed as adjusted odds ratios (AOR) (95% confidence interval).

AOR: adjusted for gestational age, small for gestational age, intraventricular hemorrhage (≥ grade 3), periventricular leukomalacia, sepsis, necrotizing enterocolitis (≥ stage 2), and retinopathy of prematurity (requiring surgery).

Respiratory morbidity rates increased from 8.8% among infants without BPD to 16.6% among those with grade 3 BPD by definition A. Rates of respiratory morbidities increased to approximately 20% and greater than 25% among those with grade 3 BPD by definitions B and C, respectively (Table 4).

**Table 4:** Mortality or morbidities due to respiratory causes at 18–24 months of corrected age.

		None	Grade 1	Grade 2	Grade 3	Total
Definition A	Death	0	0	0	8	8
	Readmission, 1	3/22	75/372	41/157	87/283	206/834
	Readmission, 2	1/22	42/372	20/157	30/283	93/834
	Readmission $, \ge$ 3	3/22	32/372	21/157	49/283	105/834
	Oxygen	0/34	0/461	0/200	2/355	2/1050
	Mechanical ventilator	0/34	0/461	0/200	1/355	1/1050
	Tracheostomy	0/34	0/461	0/200	12/355	12/1050
	Respiratory morbidities	3/34 (8.8)	32/461 (6.9)	21/200 (10.5)	59/355 (16.6)	115/1050 (11.0)
Definition B	Death	0	0	3	5	8
	Readmission, 1	85/417	47/188	33/111	41/116	206/832
	Readmission, 2	45/417	26/188	9/111	12/116	92/832
	Readmission $, \ge$ 3	39/417	24/188	19/111	23/116	105/832
	Oxygen	0/521	1/239	0/139	1/149	2/1048
	Mechanical ventilator	0/521	0/239	0/139	1/149	1/1048
	Tracheostomy	0/521	0/239	2/139	10/149	12/1048
	Respiratory morbidities	39/521 (7.5)	25/239 (10.5)	20/139 (14.4)	31/149 (20.8)	115/1048 (11.0)
Definition C	Death	0	0	3	5	8
	Readmission, 1	85/417	32/132	62/222	27/61	206/832
	Readmission, 2	45/417	20/132	22/222	5/61	92/832
	Readmission $, \ge$ 3	39/417	17/132	36/222	13/61	105/832
	Oxygen	0/521	0/168	1/277	1/82	2/1048
	Mechanical ventilator	0/521	0/168	0/277	1/82	1/1048
	Tracheostomy	0/521	0/168	4/277	8/82	12/1048
	Respiratory morbidities	39/521 (7.5)	17/168 (10.1)	38/277 (13.7)	21/82 (25.6)	115/1048 (11.0)

Values are expressed as number (%).

Respiratory morbidities: mortality or morbidities due to respiratory causes, defined as (1) death due to respiratory causes, (2) readmission (≥ 3 times) due to respiratory causes, or (3) oxygen, mechanical ventilator, or tracheostomy at 18–24 months of corrected age.

Adjusted odds ratios (AOR) [OR was adjusted for GA, SGA, intraventricular hemorrhage (IVH) (≥ grade 3), periventricular leukomalacia (PVL), sepsis, necrotizing enterocolitis (NEC) (≥ stage 2), and retinopathy of prematurity (ROP) (requiring surgery), which might affect mortality and long-term neurodevelopmental and growth outcomes] of mortality or morbidities due to respiratory causes compared to no BPD as per each definition were analyzed.

According to definition A, infants with BPD did not have a higher risk of mortality or morbidities due to respiratory causes than infants without BPD.

By definition B, when compared with infants without BPD, infants with grade 2 and 3 BPD had 2.28 times (95% confidence interval [CI] 1.25, 4.15) and 3.40 times (95% CI 1.98, 5.83) higher risk for mortality or morbidities due to respiratory causes, respectively. By definition C, when compared with infants without BPD, infants with grade 2 and 3 BPD had 2.10 times (95% CI 1.27, 3.48) and 4.41 times (95% CI 2.37, 8.23) higher risk for mortality or morbidities due to respiratory causes, respectively.

### Neurodevelopmental impairment (Table 5)

A total of 196 infants (23.4%, 196/839) had mental developmental delay, 269 infants (28.1%, 269/959) had motor developmental delay, and 83 infants (13.0%, 83/639) had social developmental delay.

**Table 5:** Adjusted odds ratios using multivariable logistic regression to evaluate neurodevelopmental impairment at 18–24 months of corrected age according to the severity of bronchopulmonary dysplasia (BPD) compared to no BPD.

		Definition A	Definition B	Definition C
Mental developmental delay	Grade 1	0.46 (0.19,1.12)	0.97 (0.62,1.54)	0.73 (0.42,1.28)
	Grade 2	0.47 (0.18,1.24)	1.58 (0.95,2.64)	1.58 (1.04,2.41)
	Grade 3	0.95 (0.38,2.39)	1.92 (1.16,3.17)	2.45 (1.30,4.61)
Motor developmental delay	Grade 1	0.62 (0.26,1.48)	0.95 (0.64,1.42)	0.98 (0.62,1.54)
	Grade 2	0.74 (0.30,1.83)	1.87 (1.19,2.95)	1.47 (1.01,2.13)
	Grade 3	1.17 (0.48,2.81)	1.84 (1.18,2.87)	2.22 (1.28,3.86)
Social developmental delay	Grade 1	0.96 (0.16,5.70)	1.45 (0.75,2.80)	1.64 (0.78,3.43)
	Grade 2	1.96 (0.32,12.13)	1.54 (0.69,3.46)	1.70 (0.91,3.20)
	Grade 3	1.83 (0.30,11.16)	2.68 (1.30,5.55)	2.42 (0.92,6.40)
Neurodevelopmental impairment	Grade 1	0.59 (0.23,1.55)	1.05 (0.67,1.64)	0.99 (0.59,1.66)
	Grade 2	0.73 (0.26,2.04)	1.91 (1.09,3.34)	1.58 (1.02,2.43)
	Grade 3	1.09 (0.40,2.98)	1.73 (1.00,3.01)	2.02 (0.93,4.40)

Values are expressed as adjusted odds ratios (AOR) (95% confidence interval).

AOR: adjusted for gestational age, small for gestational age, intraventricular hemorrhage (≥ grade 3), periventricular leukomalacia, sepsis, necrotizing enterocolitis (≥ stage 2), and retinopathy of prematurity (requiring surgery). Neurodevelopmental impairment: (1) mental developmental delay, (2) motor developmental delay, or (3) social developmental delay.

The rates of neurodevelopmental impairment were approximately 50% in grade 3 BPD by definition A. However, neurodevelopmental impairment rates increased to approximately 50% in grade 2 BPD by definitions B and C, which were greater than 50% in grade 3 BPD by definitions B and C (Table 6).

**Table 6:** Neurodevelopmental impairment at 18–24 months of corrected age.

		None	Grade 1	Grade 2	Grade 3	Total
Definition A	Mental developmental delay	8/29 (27.6)	58/368 (15.8)	29/161 (18.0)	101/281 (35.9)	196/839 (23.4)
	Motor developmental delay	8/32 (25.0)	82/418 (19.6)	47/182 (25.8)	132/327 (40.4)	269/959 (28.1)
	Social developmental delay	1/21 (4.8)	22/296 (7.4)	18/116 (15.5)	42/206 (20.4)	83/639 (13.0)
	Neurodevelopmental impairment	7/21 (33.3)	80/296 (27.0)	41/116 (35.3)	104/206 (50.5)	232/639 (36.3)
Definition B	Mental developmental delay	76/423 (18.0)	41/188 (21.8)	37/114 (32.5)	42/113 (37.2)	196/838 (23.4)
	Motor developmental delay	101/476 (21.2)	57/219 (26.0)	54/127 (42.5)	57/136 (41.9)	269/958 (28.1)
	Social developmental delay	27/336 (8.0)	22/146 (15.1)	13/78 (16.7)	21/79 (26.6)	83/639 (13.0)
	Neurodevelopmental impairment	97/336 (28.9)	54/146 (37.0)	41/78 (52.6)	40/79 (50.6)	232/639 (36.3)
Definition C	Mental developmental delay	76/423 (18.0)	21/131 (16.0)	74/227 (32.6)	25/57 (43.7)	196/838 (23.4)
	Motor developmental delay	101/476 (21.2)	39/152 (25.7)	95/257 (37.0)	34/73 (46.6)	269/958 (28.1)
	Social developmental delay	27/336 (8.0)	14/98 (14.3)	33/171 (19.3)	9/34 (26.5)	83/639 (13.0)
	Neurodevelopmental impairment	97/336 (28.9)	33/98 (33.7)	83/171 (48.5)	19/34 (55.9)	232/639 (36.3)

Values are expressed as number (%).

Neurodevelopmental impairment: (1) mental developmental delay, (2) motor developmental delay, or (3) social developmental delay.

According to definition A, infants with BPD did not have a higher risk for neurodevelopmental impairment than infants without BPD.

As per definition B, infants with grade 3 BPD had 1.92 times (95% CI 1.16, 3.17) higher risk for mental developmental delay than infants without BPD. By definition C, when compared with infants without BPD, infants with grade 2 and 3 BPD had 1.58 times (95% CI 1.04, 2.41) and 2.45 times (95% CI 1.30, 4.61) higher risk for mental developmental delay, respectively.

As per definition B, when compared with infants without BPD, infants with grade 2 and 3 BPD had 1.87 times (95% CI 1.19, 2.95) and 1.84 times (95% CI 1.18, 2.87) higher risk for motor developmental delay, respectively. As per definition C, when compared with infants without BPD, infants with grades 2 and 3 BPD had 1.47 times (95% CI 1.01, 2.13) and 2.22 times (95% CI 1.28, 3.86) higher risk for motor developmental delay, respectively. The risk was lower in grade 3 than in grade 2 as per definition B, which increased much in grade 3 BPD as per definition C compared to grade 3 BPD as per definition B.

As per definition B, when compared with infants without BPD, infants with grade 3 BPD had 2.68 times (95% CI 1.30, 5.55) higher risk of social developmental delay. By definition C, the risk of social developmental delay increased according to the severity of BPD without statistical significance.

### Growth restriction (Table 7)

A total of 391 infants (38.1%, 391/1027) had growth restriction at follow-up between 18 and 24 months of CA.

**Table 7:** Adjusted odds ratios using multivariable logistic regression to evaluate growth restriction at 18–24 months of corrected age according to the severity of bronchopulmonary dysplasia (BPD) compared to no BPD.

	Definition A	Definition B	Definition C
Grade 1	1.51 (0.63,3.60)	1.33 (0.94,1.87)	1.28 (0.87,1.88)
Grade 2	1.89 (0.77,4.66)	1.72 (1.13,2.62)	1.65 (1.18,2.30)
Grade 3	2.80 (1.15,6.81)	2.03 (1.35,3.05)	2.39 (1.43,4.01)

Values are expressed as adjusted odds ratios (AOR) (95% confidence interval).

AOR: adjusted for gestational age, small for gestational age, intraventricular hemorrhage (≥ grade 3), periventricular leukomalacia, sepsis, necrotizing enterocolitis (≥ stage 2), and retinopathy of prematurity (requiring surgery).

Growth restriction rates were approximately 50% in grade 3 BPD as per definition A. However, growth restriction rates increased to approximately 50% in grade 2 BPD by definitions B and C, which were greater than 50% in grade 3 BPD by definitions B and C (Table 8).

**Table 8:** Growth restriction defined as z-scores < − 1.28 (equivalent to < 10^th^ percentile) of weight, height, or head circumference at 18–24 months of corrected age.

		None	Grade 1	Grade 2	Grade 3	Total
Definition A	Wt	2/34 (5.9)	77/450 (17.1)	43/195 (22.1)	112/346 (32.4)	234/1025 (22.8)
	Ht	1/33 (3.0)	70/425 (16.5)	22/178 (12.4)	95/321 (29.6)	188/957 (19.6)
	HC	5/32 (15.6)	68/393 (17.3)	47/149 (31.5)	109/282 (38.7)	229/856 (26.8)
	Growth restriction	7/34 (20.6)	135/451 (29.9)	71/195 (36.4)	178/347 (51.3)	391/1027 (38.1)
	Z-scores of weight	− 0.14 ± 0.83	− 0.25 ± 1.10	− 0.50 ± 1.04	− 0.92 ± 1.18	− 0.52 ± 1.15
	Z-scores of height	0.01 ± 0.82	− 0.24 ± 1.29	− 0.21 ± 1.12	− 0.68 ± 1.29	− 0.38 ± 1.26
	Z-scores of head circumference	− 0.13 ± 1.11	− 0.29 ± 1.26	− 0.67 ± 1.38	− 1.04 ± 1.37	− 0.60 ± 1.35
Definition B	Wt	83/510 (16.3)	59/233 (25.3)	47/138 (34.1)	45/142 (31.7)	234/1023 (22.9)
	Ht	72/482 (14.9)	42/215 (19.5)	35/134 (26.1)	38/125 (30.4)	187/956 (19.6)
	HC	79/447 (17.7)	64/194 (33.0)	39/105 (37.1)	47/108 (43.5)	229/854 (26.8)
	Growth restriction	152/511 (29.8)	94/233 (40.3)	69/138 (50.0)	75/143 (52.5)	390/1025 (38.0)
	Z-scores of weight	− 0.25 ± 1.07	− 0.64 ± 1.06	− 0.89 ± 1.16	− 0.95 ± 1.28	− 0.52 ± 1.15
	Z-scores of height	− 0.20 ± 1.26	− 0.47 ± 1.12	− 0.56 ± 1.24	− 0.69 ± 1.43	− 0.37 ± 1.26
	Z-scores of head circumference	− 0.29 ± 1.26	− 0.75 ± 1.37	− 0.99 ± 1.30	− 1.20 ± 1.44	− 0.60 ± 1.36
Definition C	Wt	83/510 (16.3)	39/163 (23.9)	81/272 (29.8)	31/78 (39.7)	234/1023 (22.9)
	Ht	72/482 (14.9)	21/148 (14.2)	68/260 (26.2)	26/66 (39.4)	187/956 (19.6)
	HC	79/447 (17.7)	40/129 (31.0)	85/224 (38.0)	25/54 (46.3)	229/854 (26.8)
	Growth restriction	152/511 (29.8)	61/163 (37.4)	132/273 (48.4)	45/78 (57.7)	390/1025 (38.0)
	Z-scores of weight	− 0.25 ± 1.07	− 0.54 ± 1.11	− 0.84 ± 1.08	− 1.16 ± 1.39	− 0.52 ± 1.15
	Z-scores of height	− 0.20 ± 1.26	− 0.34 ± 1.13	− 0.55 ± 1.19	− 1.05 ± 1.52	− 0.37 ± 1.26
	Z-scores of head circumference	− 0.29 ± 1.26	− 0.65 ± 1.37	− 0.98 ± 1.34	− 1.42 ± 1.41	− 0.60 ± 1.36

Values are expressed as number (%) or mean ± standard deviation.

Wt, number of infants with z-scores <  − 1.28 of weight at 18–24 months of corrected age; Ht, number of infants with z-scores <  − 1.28 of height at 18–24 months of corrected age; HC, numbers of infants with z-scores <  − 1.28 of head circumference at 18–24 months of corrected age; Growth restriction, numbers of infants with z-scores <  − 1.28 of weight, height, or head circumference at 18–24 months of corrected age.

As per definition A, infants with grade 3 BPD had 2.80 times (95% CI 1.15, 6.81) higher risk for growth restriction than infants without BPD. As per definition B, when compared with infants without BPD, infants with grade 2 and 3 BPD had 1.72 times (95% CI 1.13, 2.62) and 2.03 times (95% CI 1.35, 3.05) higher risk for growth restriction, respectively. As per definition C, when compared with infants without BPD, infants with grade 2 and 3 BPD had 1.65 times (95% CI 1.18, 2.30) and 2.39 times (95% CI 1.43, 4.01) higher risk for growth restriction, respectively.

## Discussion

In the present study, we have clearly demonstrated that EPIs of 23–27 weeks’ gestation with BPD, according to the original NICHD 2001 definition (definition A), did not show significantly increased risks for long-term respiratory mortality, morbidities, or neurodevelopmental delay. However, EPIs with BPD diagnosed as per the modified NICHD 2016 definition (definition B) and modified Jensen 2019 definition (definition C) showed significantly increased risks for poor long-term outcomes, such as respiratory mortality and morbidities, neurodevelopmental delay, and growth restriction at 18–24 months of CA.

It has been 20 years since the introduction of the NICHD 2001 definition, which relies heavily on the concentration and duration of oxygen. Although this definition is still one of the most widely used definitions, the definition of respiratory support at 36 weeks’ PMA regardless of oxygen or respiratory support during the first 28 days of life has recently emerged as one of the most commonly used definitions^8,17^. In fact, Hines et al. revealed, through a systemic review, that the definition of BPD as respiratory support at 36 weeks’ PMA was the most commonly used in 45% of studies, followed by the NICHD 2001 definition in 30% of studies^17^.

Various NIVs have been developed, and respiratory management strategies have been changed. Infants on high-flow nasal cannula cannot be classified according to the NICHD 2001 definition. In addition, severe BPD, according to the NICHD 2001 definition, constitutes a heterogeneous group. The respiratory status of infants receiving only oxygen by nasal cannula and infants requiring mechanical ventilation at 36 weeks’ PMA are significantly different. However, they are grouped in the same category of “severe BPD” according to the NICHD 2001 definition. Severe BPD, according to the NICHD 2001 definition, has to be sub-classified to better define disease severity^18^. We also have to consider the improved survival of EPIs with GA < 28 weeks who require oxygen in the first few weeks of life.

BPD, as per definition A, was not associated with respiratory morbidity or neurodevelopmental impairment in this study. Only grade 3 BPD as per definition A was associated with growth restriction at 18–24 months of CA. Grade 1 BPD as per definition A with oxygen dependency during the first 28 days of life is not very important for these tiny preterm infants with a GA of 23–27 weeks. Almost all grade 1 BPD cases as per definition A were assigned to no BPD according to definitions B and C in this study. Over 90% of preterm infants with GA of 23–27 weeks had BPD according to definition A. According to a meta-analysis by Gou et al., oxygen dependency in the first 28 days of life (mild BPD by the NICHD 2001 definition) was not associated with cerebral palsy^10^. In addition, preterm infants with grade 1 BPD as per definition A did not increase the risk of long-term morbidities in this study, so there is no need to classify those patients as “BPD”.

Definition B in this study is based on the use of oxygen and positive pressure only at 36 weeks’ PMA^13^. Grades 2 and 3 BPD by definition B increased respiratory morbidities, motor developmental delay, and growth restriction in this study. However, the most important drawback of definition B is that it is too complicated for clinical application. Patients were assigned to different levels of grade according to the oxygen concentration, although the same flow rates were used by the NICHD 2016 definition. Therefore, the NICHD 2016 definition had to be modified to definition B because of the complexity of the subdivided oxygen concentration and flow rate.

Definition C in this study is based on the use of positive pressure at 36 weeks’ PMA instead of supplemental oxygen, which was modified from the new definition provided by Jensen et al. in 2019. The Jensen 2019 definition predicted mortality or morbidities due to respiratory cause in 81% and mortality or neurodevelopmental impairment in 69% of preterm infants with GA < 32 weeks between 18 and 26 months of CA^14^. Grades 2 and 3 BPD, as per definition C, significantly increased respiratory morbidities, mental developmental delay, motor developmental delay, and growth restriction in this study. We found a stepwise increment in the risks for these long-term morbidities according to the severity of BPD defined as per definition C. Following definition C, more than four-fold higher risk of respiratory morbidities and more than two-fold higher risk of mental and motor developmental delay and growth restriction were found among infants with grade 3 BPD compared to those without BPD. These findings strongly support the clinical usefulness of the classification of BPD severity by definition C. In addition, definition C is very simple and easy to utilize clinically in most preterm infants.

Mortality or morbidities due to respiratory causes included readmission ≥ 3 times due to respiratory causes after discharge from the NICU prior to follow-up in this study. This definition was similar to those reported by the Canadian Neonatal Follow-Up Network^19^ and Isayama et al.^20^. The definition of respiratory morbidities by Jensen et al.^14^ was similar; however, they also included continued hospitalization due to respiratory causes at or beyond 50 weeks’ PMA. In this study, 234 infants among 527 preterm infants with BPD (234/527 [44.4%], defined as B or C) were readmitted at least once during 18–24 months of CA. This result is similar to that of Thébaud et al., who reported that about 50% of preterm infants with BPD were readmitted during the first 2 years of life^21^.

Despite remarkable advances in perinatal and neonatal care and improved survival of EPIs, the incidence of BPD has not decreased or even increased^1,22^. The overall incidence of BPD diagnosed as requiring oxygen or respiratory support at 36 weeks’ PMA was reported to be approximately 40% to 45% in preterm infants with GA < 29 weeks^23,24^. Walsh et al. reported that among 45 VLBWIs with BPD diagnosed with oxygen dependency at 36 weeks’ PMA, 15 infants successfully ceased oxygen supplementation, as per the oxygen reduction test, and were hence classified as no BPD^25^. This result reflects that the need for oxygen is somewhat determined by the attending physician’s care practice, not by physiologic assessment. Inter-center variability in oxygen supply can affect the incidence and severity of BPD. This is a limitation of the currently used definition of BPD, which is based on the use of oxygen. Therefore, definition C, based on the positive pressure instead of oxygen, is more reasonable than definitions A or B.

Jensen et al. suggested that the optimal BPD definition predicted respiratory morbidities better than neurodevelopmental impairment^14^. Similarly, Linsell et al. reported that these results are quite natural, because diverse factors, in addition to respiratory morbidities, contribute to adverse neurodevelopment outcomes in these very preterm infants^26^. Mental and motor developmental delay was increased stepwise according to the severity of BPD as per definition C in preterm infants with GA < 28 weeks in this study. Jensen et al. reported that death or neurodevelopmental impairment increased according to the severity of BPD in preterm infants with GA < 32 weeks (33% with no BPD, 46% with grade 1, 60% with grade 2, and 79% with grade 3 BPD)^14^. Moreover, prolonged respiratory support is associated with an increased risk of mortality or neurodevelopmental disability, which implies an important stepwise relationship between the severity of BPD and poor neurodevelopmental outcome^27^.

There are some limitations to the present study. First, since we used the KNN pre-set registry data, some modifications from the original description of the NICHD 2016 definition or Jensen 2019 definition in definitions B and C, respectively, were inevitable, which can cause some difficulty in directly comparing the results with other studies that used the original definitions. Second, there is heterogeneity in long-term neurodevelopmental studies conducted among enrolled infants because of the multi-center nature of the registry. We prepared composite data, to use as much data as possible and to minimize selection bias; nevertheless, bias may still persist because of inter-hospital variability or inter-test variability, including recall bias arising from parent questionnaires, such as Korean Developmental Screening Test for Infants and Children (K-DST) or Korean Ages & Stages Questionnaires (K-ASQ). Furthermore, follow-up rates were highly variable with inter-hospital variability. High NICU volume with greater number of personnel and resources, easy approaches to follow-up in clinics, younger GA (≤ 29 weeks), and BWt (≤ 1,160 g) were associated with higher follow-up rates^28^.

However, a strength of this study was the use of a nationwide, prospective cohort registry covering > 80% of VLBWIs in Korea with good data management^29^; further, there was no racial difference, which can exclude various potential biases. Moreover, the subjects of this study were EPIs of 23–27 weeks of gestation, who are the most vulnerable and at high risk of BPD, and have poor respiratory, neurodevelopmental, and growth outcomes compared to more mature preterm infants.

In conclusion, EPIs with grade 3 BPD as per the modified NICHD 2016 definition had a 3.40-fold higher risk for mortality or morbidities due to respiratory cause, 1.92-fold higher risk for mental developmental delay, 1.84-fold higher risk for motor developmental delay, 2.68-fold higher risk for social developmental delay, and 2.03-fold higher risk for growth restriction than those without BPD. EPIs with severe BPD, dependent on IV (grade 3 BPD in modified Jensen 2019 definition), had a 4.41-fold higher risk for mortality or morbidities due to respiratory cause, a 2.45-fold higher risk for mental developmental delay, a 2.22-fold higher risk for motor developmental delay, and a 2.39-fold higher risk for growth restriction than those without BPD. Moderate or severe BPD defined as per the original NICHD 2001 BPD definition was no longer associated with an increased risk of long-term respiratory or neurodevelopmental poor outcome in EPIs. Therefore, more recently suggested definitions of BPD, at least in most immature infants at high risk of BPD, such as EPIs, need to be adopted., which mainly classify BPD by the type of respiratory support required, to identify more high-risk infants with poor long-term outcomes requiring early intervention.

## Methods

The KNN is a nationwide, prospective cohort registry of VLBWIs admitted to the 70 participating NICUs, covering > 80% of VLBWIs in Korea since it was launched in 2013^29^. Data for a total of 2220 VLBWIs born between January 1, 2013 and December 31, 2015, with a GA of 23–27 weeks, who were registered in the KNN were collected. A total of 512 infants who died before 36 weeks PMA and one infant who died due to major congenital abnormalities were excluded. Therefore, a total of 1707 VLBWIs with GA of 23–27 weeks were registered, and among them, 1050 were enrolled in the present study who were followed up at 18–24 months of CA.

BPD was assessed at 36 weeks’ PMA, and infants were classified according to the severity of BPD as no, grade 1, grade 2, and grade 3 BPD by definition. The risk of poor outcomes of respiratory, neurodevelopmental, and growth at 18–24 months of CA in infants with BPD were compared with those in infants without BPD for each definition. Definition A is the original NICHD 2001 definition^9^ with the precondition of oxygen or respiratory support in at least the first 28 days of life and assessed at 36 weeks’ PMA. Grade 1 (mild) BPD was defined as breathing room air, grade 2 (moderate) BPD as need for < 30% oxygen, and grade 3 (severe) BPD as need for ≥ 30% oxygen or positive pressure. KNN originally collected data using the NICHD 2001 definition for BPD. Definition B is the modified NICHD 2016 definition^13^. There is no precondition for oxygen or respiratory support for at least the first 28 days of life. BPD was assessed only at 36 weeks’ PMA according to oxygen concentration (0.21, 0.22–0.29, or ≥ 0.3) and respiratory support (none, NIV, or IV). NIV in this study included nasal cannula ≥ 2 L/min, nasal continuous positive airway pressure (N-CPAP), or noninvasive positive pressure ventilation (NIPPV). Grade 1 included all infants with a nasal cannula < 2 L/min or NIV with FiO_2_ 0.21. Grade 2 included NIV with FiO_2_ 0.22–0.29 or IV with FiO_2_ 0.21. Grade 3 included NIV with FiO_2_ ≥ 0.3 or IV with FiO_2_ > 0.21. The differences between definition B adopted in this study and the NICHD 2016 definition were as follows: (1) NIV included nasal cannula ≥ 2 L/min in definition B; however, it is ≥ 3 L/min in the NICHD 2016 definition; (2) patients with nasal cannula < 2 L/min were allocated to grade 1 in definition B; however, nasal cannula < 1 L/min with FiO_2_ > 0.7 were allocated to grade 2 according to the NICHD 2016 definition (Supplementary Table 3). Definition C is the modified Jensen 2019 definition^14^. There is no precondition of oxygen or respiratory support for at least the first 28 days of life, nor subdivision by oxygen concentration. BPD was graded by respiratory support assessed at 36 weeks’ PMA. Grade 1 included nasal cannula < 2 L/min, grade 2 included NIV (included nasal cannula ≥ 2 L/min), and grade 3 included IV. The difference between definition C in this study and Jensen 2019 definition was NIV, which included nasal cannula ≥ 2 L/min in definition C and > 2 L/min in Jensen 2019 definition (Supplementary Table 4).

Respiratory status, neurodevelopment, and growth were assessed at follow-up between 18 to 24 months of CA. Mortality or morbidities due to respiratory causes were defined as (1) death due to respiratory causes between 36 weeks’ PMA and 18–24 months of CA; (2) readmission (≥ 3 times) due to respiratory cause after discharge from the NICU prior to follow-up; or (3) oxygen, mechanical ventilator, or tracheostomy at follow-up.

Neurodevelopmental assessments included composite scores on the Bayley Scales of Infant Development-Second Edition (BSID II)^30^, BSID III^31^, K-ASQ^32^, and K-DST^33^. Different neurodevelopmental assessment modalities were used with inter-hospital variability, including the BSID II, BSID III, K-ASQ, or K-DST, because of the multi-center registry nature of this study. K-ASQ and K-DST are questionnaires, not confirmatory tests. To use as much data as possible, in order to minimize selection bias, composite data of each domain in each modality were developed. BSID II has two domains (mental and psychomotor), and BSID III has three domains (cognitive, language, and motor). However, the K-ASQ has five domains, and the K-DST has six domains, including social development. Therefore, we developed the composite data as follows. (1) Mental domain in BSID II; (2) cognitive and language domains in BSID III; (3) communication and problem-solving domains in K-ASQ; and (4) cognition, language, and self-help domains in the K-DST were used to evaluate mental development. The motor domain in BSID II and BSID III, gross motor, and fine motor domains in the K-ASQ and K-DST were used to evaluate motor development. The personal-social domain in the K-ASQ and sociality domain in the K-DST were used to evaluate social development. The K-ASQ has been used in Korea since 2000 for developmental assessment of infants^34^, which is a revised Korean version of the ASQ developed in the US by Squires^32^. The K-ASQ includes five domains: gross motor, fine motor, communication, problem-solving, and personal-social. The K-ASQ showed relatively high concurrent validity with DDST II, which can be used for screening and follow-up of developmental delay^35^. The K-DST has been used for developmental screening of infants and children in Korea since 2014 as part of a national health screening program by the Korean government. The K-DST includes six domains: gross motor, fine motor, cognition, language, sociality, and self-help. The specificity of the K-DST compared to the BSID II is > 70% in preterm infants, and it could be a useful screening tool for neurodevelopmental assessments^36^.

Mental developmental delay was defined as (1) a mental developmental index < 70 on the BSID II; (2) cognitive or language < 70 on the BSID III; (3) communication or problem-solving score less than the cut-off value on the K-ASQ; or (4) cognition, language, or self-help score is less than the cut-off value on the K-DST. Motor developmental delay was defined as (1) a psychomotor developmental index < 70 on the BSID II; (2) motor < 70 on the BSID III; (3) gross motor or fine motor score is less than the cut-off value on K-ASQ; (4) gross motor or fine motor score is less than the cut-off value on K-DST; or (5) Cerebral palsy defined as Gross Motor Functional Classification System ≥ 2^37^. Social developmental delay was defined as (1) a personal-social score less than the cut-off value on the K-ASQ, or (2) sociality is less than the cut-off value on the K-DST.

Neurodevelopmental impairment was defined as (1) mental developmental delay, (2) motor developmental delay, or (3) social developmental delay.

Growth restriction was defined as failure of catch-up growth, defined as z-scores < − 1.28 (equivalent to < 10th percentile) of weight, height, or head circumference according to the CA by the 2006 World Health Organization (WHO) Child Growth Standards^38^.

### Statistical analysis

Data are presented as mean ± standard deviation for continuous variables and as number (percentage) for categorical variables. The chi-square test or Fisher’s exact test was performed for categorical variables, and the t-test or Mann–Whitney U test was used for continuous variables, as appropriate. ORs were adjusted for GA, SGA, IVH (≥ grade 3), PVL, sepsis, NEC (≥ stage 2), and ROP (requiring surgery), which might affect mortality, long-term neurodevelopmental, and growth outcomes. AOR and 95% CI compared to no BPD according to the severity of BPD using multivariable logistic regression were presented to evaluate increased risks of respiratory morbidities, neurodevelopmental impairment, or growth restriction. In each model, the study outcome was considered as a binary dependent variable, and the BPD definition was considered as a categorical independent variable. All statistical analyses were performed using SAS (version 9.4; SAS Inc., Cary, NC, USA). Statistical significance was set at *p* < 0.05.

### Ethics statement

The institutional review board of each participating center approved the KNN data registry during admission and follow-up, and written informed consent was obtained from the infants’ parents at enrollment. All the methods were performed in accordance with the approved protocol.

The names of the institutional review board of the KNN participating hospitals were as follows: The institutional review board of Gachon University Gil Medical Center, The Catholic University of Korea Bucheon ST. Mary's Hospital, The Catholic University of Korea Seoul ST. Mary's Hospital, The Catholic University of Korea ST. Vincent's Hospital, The Catholic University of Korea Yeouido ST. Mary's Hospital, The Catholic University v of Korea Uijeongbu ST. Mary's Hospital, Gangnam Severance Hospital, Kyung Hee University Hospital at Gangdong, GangNeung Asan Hospital, Kangbuk Samsung Hospital, Kangwon National University Hospital, Konkuk University Medical Center, Konyang University Hospital, Kyungpook National University Hospital, Gyeongsang National University Hospital, Kyung Hee University Medical center, Keimyung University Dongsan Medical Center, Korea University Guro Hospital, Korea University Ansan Hospital, Korea University Anam Hospital, Kosin University Gospel Hospital, National Health Insurance Service Iilsan Hospital, Daegu Catholic University Medical Center, Dongguk University Ilsan Hospital, Dong-A University Hospital, Seoul Metropolitan Government-Seoul National University Boramae Medical Center, Pusan National University Hospital, Busan ST Mary's Hospital, Seoul National University Bundang Hospital, Samsung Medical Center, Samsung Changwon Medical Center, Seoul National University Hospital, Asan Medical Center, Sungae Hospital, Severance Hospital, Soonchunhyang University Hospital Bucheon, Soonchunhyang University Hospital Seoul, Soonchunhyang University Hospital Cheonan, Ajou University Hospital, Pusan National University Children's Hospital, Yeungnam University Hospital, Ulsan University Hospital, Wonkwang University School of Medicine & Hospital, Wonju Severance Christian Hospital, Eulji University Hospital, Eulji General Hospital, Ewha Womans University Medical Center, Inje University Busan Paik Hospital, Inje University Sanggye Paik Hospital, Inje University Ilsan Paik Hospital, Inje University Haeundae Paik Hospital, Inha University Hospital, Chonnam National University Hospital, Chonbuk National University Hospital, Cheil General Hospital & Women's Healthcare Center, Jeju National University Hospital, Chosun University Hospital, Chung-Ang University Hospital, CHA Gangnam Medical Center, CHA University, CHA Bundang Medical Center, CHA University, Chungnam National University Hospital, Chungbuk National University, Kyungpook National University Chilgok Hospital, Kangnam Sacred Heart Hospital, Kangdong Sacred Heart Hospital, Hanyang University Guri Hospital, and Hanyang University Medical Center.

## Supplementary Information

Supplementary Information.
