# Supplementary material for: Definitions of bronchopulmonary dysplasia and long-term outcomes of extremely preterm infants in Korean Neonatal Network
Source: Sci Rep. 2021 Dec 21;11:24349. doi: 10.1038/s41598-021-03644-7 (PMC8692520; doi:10.1038/s41598-021-03644-7)
Supplement: Supplementary file 1 — Supplementary Information. [file 41598_2021_3644_MOESM1_ESM.docx]

**Table 1. Characteristics of initially identified patients who were registered in the Korean Neonatal Network.**

|  | **Total**  N=1,707 |
| --- | --- |
| Gestational age, weeks | 25.7±1.2 |
| 23, n (%) | 92/1,707 (5.4) |
| 24, n (%) | 200/1,707 (11.7) |
| 25, n (%) | 390/1,707 (22.8) |
| 26, n (%) | 462/1,707 (27.1) |
| 27, n (%) | 563/1,707 (33.0) |
| Birth weight, g | 875.5±193.7 |
| <500g, n (%) | 43/1,707 (2.5) |
| 500~<750g, n (%) | 411/1,707 (24.1) |
| 750~<1,000g, n (%) | 783/1,707 (45.9) |
| 1,000~<1,500g, n (%) | 470/1,707 (27.5) |
| Male, n(%) (sex) | 883/1,707 (51.7) |
| Small for gestational age, n (%) | 112/1,707 (6.6) |
| Apgar score at 1 min | 3.8±1.8 |
| Apgar score at 5 min | 6.1±1.8 |
| Antenatal steroid therapy, n (%) | 1369/1,676 (81.7) |
| Maternal chorioamnionitis | 685/1,447 (47.3) |
| Maternal GDM | 96/1,695 (5.7) |
| Maternal PIH | 144/1,684 (8.6) |
| Follow-up at 18–24 months corrected age, n (%) | 1,050/1,481 (70.9) |

Values are expressed as mean ± standard deviation or number (%). GDM, gestational diabetes mellitus; PIH, pregnancy induced hypertension.

**Table 2. Agreement between three different definitions of bronchopulmonary dysplasia.**

| Definition A | Definition B | | | | Definition C | | | |  |
| --- | --- | --- | --- | --- | --- | --- | --- | --- | --- |
|  | No | Grade 1 | Grade 2 | Grade 3 | No | Grade 1 | Grade 2 | Grade 3 | Total |
| No | 34 | 0 | 0 | 0 | 34 | 0 | 0 | 0 | 34 |
| Grade 1 | 455 | 6 | 0 | 0 | 455 | 5 | 1 | 0 | 461 |
| Grade 2 | 21 | 160 | 18 | 1 | 21 | 158 | 21 | 0 | 200 |
| Grade 3 | 11 | 73 | 121 | 148 | 11 | 5 | 255 | 82 | 353 |
| Total | 521 | 239 | 139 | 149 | 521 | 168 | 277 | 82 | 1,048 |

The differences between definitions are described in Supplementary Tables 6 and 7.

| **Table 3. Differences between NICHD 2016 definition and definition B.**   \| NICHD 2016 definition \| \| \| \| \| \| \| --- \| --- \| --- \| --- \| --- \| --- \| \| Mode of respiratory support \| \| O_2_ <1L/min \| O_2_ 1-<3L/min, Hood O_2_ \| O_2_ ≥3L/min, NIV \| IV \| \| FiO_2_ \| 0.21 \| - \| - \| Grade 1 \| Grade 2 \| \| 0.22–0.29 \| Grade 1 \| Grade 1 \| Grade 2 \| Grade 3 \| \| ≥0.3 \| Grade 1 (Grade 2 >0.7) \| Grade 2 \| Grade 3 \| Grade 3 \| \| Definition B \| \| \| \| \| \| \| mode of respiratory support \| \| O_2_ <2L/min \| \| O_2_ ≥2L/min, NIV \| IV \| \| FiO_2_ \| 0.21 \| No BPD \| \| Grade 1 \| Grade 2 \| \| 0.22–0.29 \| Grade 1 \| \| Grade 2 \| Grade 3 \| \| ≥0.3 \| - \| \| Grade 3 \| Grade 3 \| |
| --- | --- | --- | --- | --- | --- | --- | --- | --- | --- | --- | --- | --- | --- | --- | --- | --- | --- | --- | --- | --- | --- | --- | --- | --- | --- | --- | --- | --- | --- | --- | --- | --- | --- | --- | --- | --- | --- | --- | --- | --- | --- | --- | --- | --- | --- | --- | --- | --- | --- | --- | --- | --- | --- | --- | --- | --- |

NIV, non-invasive ventilator; IV, invasive ventilator.

Oxygen concentration was classified as 0.21, 0.22–0.69, and ≥0.7, with nasal cannula <1 L/min; 0.21, 0.22–0.29, and ≥0.3 with nasal cannula ≥1 L/min or ventilator in the NICHD 2016 definition. However, oxygen concentration was classified as 0.21, 0.22–0.29, and ≥0.3, regardless of the flow rate in the Korean Neonatal Network (KNN) registry. The flow rate of the nasal cannula was classified as <1 L/min, 1–<3 L/min, and ≥3 L/min according to the NICHD 2016 definition. However, the flow rate of the nasal cannula was divided into <2 L/min and ≥2 L/min in the KNN registry.

**Table 4. Differences between Jensen 2019 definition and definition C.**

| Jensen 2019 definition | | | |
| --- | --- | --- | --- |
| Room air | O_2_ **≤**2L/min | NIV (O_2_ **>**2L/min) | IV |
| No BPD | Grade 1 | Grade 2 | Grade 3 |
| Definition C | | | |
| Room air | O_2_ **<**2L/min | NIV (O_2_ **≥**2L/min) | IV |
| No BPD | Grade 1 | Grade 2 | Grade 3 |

NIV, non-invasive ventilator; IV, invasive ventilator.

NIV includes nasal cannula >2 L/min according to the Jensen 2019 definition, and ≥2 L/min in definition C.
